# Environmental Radionuclides in Surface Soils of Vietnam

**DOI:** 10.1100/tsw.2002.181

**Published:** 2002-04-26

**Authors:** P.D. Hien, H.T. Hiep, N.H. Quang, T.V. Luyen, N.T. Binh, N.T. Ngo, N. Q. Long, V. T. Bac

**Affiliations:** Vietnam Atomic Energy Agency, 59 Ly Thuong Kiet, Hanoi, Vietnam

**Keywords:** and ^137^Cs in undisturbed soils, gamma spectrometry, absorbed dose in air, relationship of ^137^Cs deposition density to latitude and annual rainfall

## Abstract

A database on U, Th, K, and Cs in surface soils was established to provide inputs for the assessment of the collective dose to the population of Vietnam and to support soil erosion studies using Cs as a tracer. A total of 292 soil samples was taken from undisturbed sites across the territory and the concentrations of radionuclides were determined by gamma spectrometry method. The multiple regression of Cs inventories against characteristics of sampling locations allowed us to establish the distribution of Cs deposition density and its relationship with latitude and annual rainfall. The Cs deposition density increases northward and varies from 178 Bq m to 1,920 Bq m. High rainfall areas in the northern and central parts of the country have received considerable Cs inputs exceeding 600 Bq m, which is the maximum value that can be expected for Vietnam from the UNSCEAR global pattern. The mean activity concentrations of naturally occurring radionuclides U, Th, and K are 45, 59, and 401 Bq kg, respectively, which entail an average absorbed dose rate in air of 62 nGy h, which is about 7% higher than the world average.

## INTRODUCTION

Gamma spectrometry analysis of soil samples can provide activity concentrations of naturally occurring radionuclides (NOR) such as $^{40}K$ and members of the U and Th series, as well as the nuclear test–derived $^{137}Cs$. About 55% of the collective dose associated with external exposure to the world population is due to terrestrial gamma rays from the NORs[1]. Meanwhile, $^{137}Cs$ is a leading component of the residual global radiation background caused by past atmospheric nuclear tests during the 1950s and early 1960s. So far, the basic input for the assessment of dose associated with past nuclear tests has been the global distribution of $^{90}Sr$ deposition density established in the 1960s and given in the UNSCEAR publication of 1969[2]. Direct gamma spectrometry measurements of $^{137}$Cs deposition density are needed to refine the database on nuclear testing fallout radionuclides.

On the other hand, $^{137}$Cs has been successfully used as a tracer in soil erosion and sedimentation studies (see [3] and references therein). So far, most studies have been carried out in the mid-latitude regions, while only a few reports appeared from equatorial areas where the nuclear-test fallout deposition is minimum[2].

Soil erosion and sedimentation studies are currently being carried out in Vietnam[4], but the assessment of the total collective dose to the population has yet to be undertaken. To support these activities, a comprehensive database of NORs and $^{137}$Cs in surface soils was thought to be necessary. For this purpose, activity concentrations (in Bq kg$^{-1}$) of NORs and inventories (in Bq m$^{-2}$) of $^{137}$Cs were measured for 292 soil samples collected across a 320,000-km$^2$ territory of Vietnam extending from 9°N to 23°N along the West Pacific coast.

## MATERIALS AND METHODS

To obtain the distribution of $^{137}$Cs deposition density (in Bq m$^{-2}$) from inventory measurements, soil samples were collected from undisturbed sites where soil erosion or accretion are unlikely to occur. Soil cores were taken to a 30-cm depth in grass-covered terrains. Mountainous and remote areas were skipped. On the 0.5° × 0.5° grid, only 100 out of 140 grid cells were sampled, and the average number of samples per grid cell was 2.9 ± 1.6. The materials were dried at 105°C for 24 h, gently grounded to powder, and sieved to pass a 2-mm mesh prior to gamma spectrometry measurements.

Activity concentrations were measured using four low-background gamma spectrometers with active volumes of the HP-Ge detectors ranging from 90 to 140 cm$^3$ and peak resolutions (FWHM) of around 1.5 keV at 662 keV. The precision of gamma spectrometry analysis of $^{238}$U, $^{232}$Th, $^{40}$K, and $^{137}$Cs, calculated as one standard error of the net area of the respective photopeak, was from 5 to 20%. The measured values and relevant characteristics of sampling sites are summarized in Table 1.

## NATURALLY OCCURRING RADIONUCLIDES

The mean concentrations of $^{238}$U, $^{232}$Th, and $^{40}$K in soils are 45, 59, and 401 Bq kg$^{-1}$, respectively (Table 1), which are higher than the corresponding world averages of 40, 40, and 370 Bq kg$^{-1}$[5]. The mean absorbed dose rate in air at 1 m above ground surface calculated, using the conversion coefficients given[1], is 63 nG h$^{-1}$, which is 7% higher than the world average.

## DISTRIBUTION OF $^{137}$Cs DEPOSITION DENSITY

The measured $^{137}$Cs inventories in soils showed increasing trends with latitude and annual rainfall of sampling locations. The multiple regression method was applied to establish these relationships. The inventory values were logarithmically transformed to create a dependent variable. Independent variables included longitude, latitude, and annual rainfall at the sampling locations. The logarithmic transformation of inventory values allowed us to achieve high goodness-of-fits ($R^2$) of the regression models; its physical interpretation is discussed below. With

**TABLE 1**
**Summary Statistics of Measured Data**

| | Mean | Stand. dev. | Median | Range |
|---|---|---|---|---|
| Latitude, °N | | | | 9.2 – 23 |
| Longitude, °E | | | | 103 – 110 |
| $^{238}U$, Bq $kg^{-1}$ | 45 | 32 | 38 | 3 – 350 |
| $^{232}Th$, Bq $kg^{-1}$ | 59 | 33 | 53 | 5 – 220 |
| $^{40}K$, Bq $kg^{-1}$ | 401 | 354 | 322 | 3 – 1,996 |
| $^{137}Cs$ activity concentration, Bq $kg^{-1}$ | 2.03 | 2.20 | 1.58 | 0.5 – 18.0 |
| $^{137}Cs$ inventory, Bq $m^{-2}$ | 643 | 384 | 590 | 129 – 3,294 |
| South Vietnam, <16°N | 441 | 315 | 361 | 129 – 1,603 |
| North Vietnam, >16°N | 808 | 359 | 750 | 314 – 3,294 |
| Bulk density, kg $m^{-3}$ | 1.22 | 0.30 | 1.23 | 0.43 – 2.1 |
| Mean annual rainfall, m | 1.97 | 0.56 | 1.88 | 0.79 – 3.8 |

*Note:* Total number of samples: 292

significance level set at $p < 0.01$, only latitude (L) and annual rainfall (AR) appeared as determinants in the regression model:

$$\mathrm{Ln(I)} - \varepsilon = (3.53 \pm 0.09) + (0.092 \pm 0.004)\mathrm{L} + (0.62 \pm 0.03)\mathrm{AR} \quad (1)$$

The two variables L and AR could explain 76% ($R^2 = 0.76$) of the total variance of Ln(I), leaving the 24% remaining variance to the residual $\varepsilon$. The latter follows an approximately normal distribution with a zero mean ($\bar{\varepsilon} = 0$) and a standard deviation of 0.30, which is apparently greater than that associated with experimental errors. The contribution from experimental errors in $\varepsilon$ can be estimated as $\mathrm{Ln}(1 + \alpha) \sim 0.1$, where $\alpha$ is the typical relative standard error of the inventory measurements ($\alpha \sim 10\%$).

The regression model (Eq. 1) can be interpreted as follows: If all soil samples were actually taken from undisturbed sites, the inventory measurements would yield the pattern of $^{137}Cs$ deposition density (D). Within the territory of Vietnam, the nuclear-test fallout deposition density varies insignificantly with longitude, as can be seen from the global pattern of $^{90}Sr$ deposition density. Therefore, only two factors control the spatial variability of the fallout deposition rate: the atmospheric concentration of $^{137}Cs$, which is mainly a function of latitude, and the amount of annual rainfall; both refer to the period of nuclear tests in the 1950s and early 1960s. The product relationship of the deposition rate with these two factors results in a linear relationship of the logarithmic deposition density with the latitude and annual rainfall. We would have in this case:

$$\mathrm{Ln(I)} - \varepsilon_{exp} = \mathrm{Ln(D(L, AR))} \quad (2)$$

where the residuals $\varepsilon_{exp}$ come from experimental errors; that is, $\varepsilon_{exp} \sim \mathrm{Ln}(1 + \alpha) \sim 0.1$. As a matter of fact, $|\varepsilon|$ in model (Eq. 1) is apparently greater than $\varepsilon_{exp}$. Therefore, besides measurement errors, there are other sources contributing to the residual term in Eq. 1. One potential source is associated with redistribution processes involving erosion, transport, and accretion of surface soil that have been taking place and causing either loss or gain of $^{137}Cs$ at the sampling locations[3]. Hence the measured inventory I was either smaller or greater than the deposition density D. It is natural, however, to assume that either case was equally likely to occur

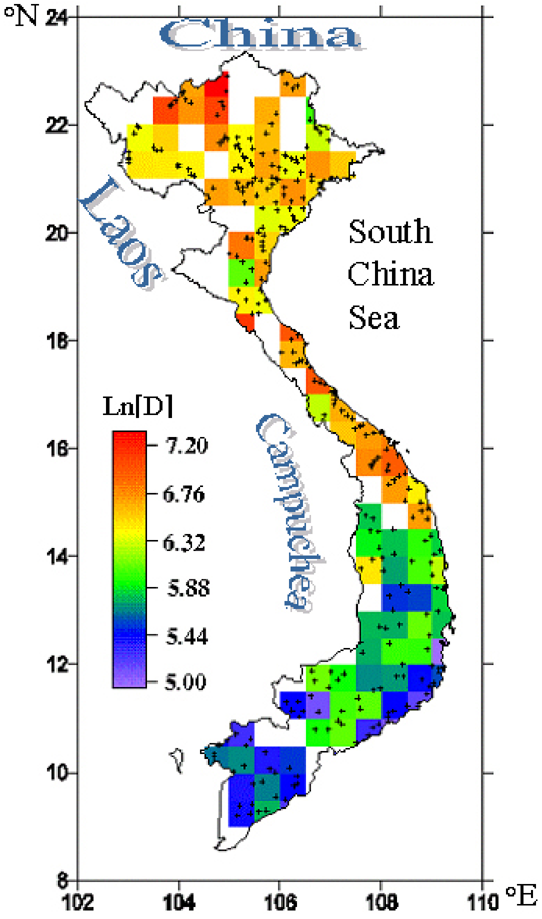

**FIGURE 1**. 0.5° × 0.5° longitude latitude gridded map of $^{137}Cs$ logarithmic deposition density.

in our sampling campaign, so that the mean logarithmic inventory for sampling locations having similar L and AR tends to converge toward the logarithmic deposition density Ln(D(L, AR)). A second source giving rise to the residual in Eq. 1 is associated with errors of the AR values. An error of 0.16 m in AR, for example, would lead to a 10% relative deviation of the deposition density predicted by Eq. 1. Thus, the multiple regression model can be rewritten as follows:

$$\mathrm{Ln(I)} - \varepsilon = \mathrm{Ln(D(L,AR))} \qquad (1')$$

where the residual $\varepsilon$ has three components associated with redistribution processes, inventory measurement uncertainty, and errors in annual rainfall. The dispersion of experimental values Ln(I) around Ln(D) is about ±0.30. This means that in ordinary scale, the typical relative deviation of the measured inventory from the deposition density is about ±35%

The effect of $^{137}Cs$ redistribution processes leading to the deviation of the measured inventory from the deposition density would be reduced if experimental data were averaged over grid cells containing many sampling points. The regression of the mean of Ln(I) upon mean characteristics of the sampling locations would then yield the same relationship as in Eq. 1 with reduced residuals. If, for example, experimental data were averaged over 0.5° × 0.5° grid cells (Fig. 1), the regression model for the grid cell inventories would be:

$$[\mathrm{Ln(I)}]_{g.c.} - \varepsilon' = (3.51 \pm 0.11) + (0.093 \pm 0.005)[L]_{g.c.} + (0.61 \pm 0.04)[AR]_{g.c.} \qquad (3)$$

where the symbol $[...]_{g.c.}$ denotes the grid-cell average. The intercept and regression coefficients in Eq. 3 remain almost similar to Eq. 1, but the new residuals $\varepsilon'$ have a standard deviation of 0.17, which is much less than $\varepsilon$ in the original model (Eq. 1). Accordingly, the regression model in Eq. 3 could explain 88% of the variance in $[Ln(I)]_{g.c.}$ across 100 grid cells. The right hand side of Eq. 3 now represents the average of logarithmic deposition density in each grid cell $[Ln(D)]_{g.c.}$.

The distribution of $[Ln(D)]_{g.c.}$ over the territory is mapped in Fig. 1, which clearly shows the annual rainfall variability superimposed on the northward increasing trend of nuclear-test fallout deposition density. The $^{137}Cs$ deposition density increases northward from 178 to 1,920 Bq $m^{-2}$; this range is larger than can be expected for Vietnam from the global pattern[2], which is from 300 to 600 Bq $m^{-2}$. Large areas in the southern part of the territory have received the $^{137}Cs$ inputs less than 300 Bq $m^{-2}$, while the deposition density in many areas with high rainfalls of the northern and central parts considerably exceeds 600 Bq $m^{-2}$.

The $^{137}Cs$ deposition density calculated by Eq. 1 agrees very well with $^{137}Cs$ reference values measured in experiments on soil erosion and sedimentation processes at some drainage basins in Lam dong province, South Vietnam[6]. Eq. 1 was also used to provide $^{137}Cs$ reference values for estimating soil erosion rate at an afforestation area of the 2,000-MW hydropower station in Hoa binh, North Vietnam[4].
